# Draft Genome Sequence of Brazilian *Escherichia coli* Uropathogenic Strain E2

**DOI:** 10.1128/genomeA.01085-16

**Published:** 2016-10-06

**Authors:** Thiago G. S. Paim, Luiza Pieta, Janira Prichula, Gustavo E. Sambrano, Renata Soares, Juliana Caierão, Jeverson Frazzon, Pedro A. d’Azevedo

**Affiliations:** a Laboratory of Molecular Microbiology, Federal University of Health Sciences of Porto Alegre, Porto Alegre, Brazil; b Institute of Food Sciences and Technology, Federal University of Rio Grande do Sul, Porto Alegre, Brazil

## Abstract

*Escherichia coli* is a common pathogen recovered from cystitis infections. In this report, we announce the draft genome sequence of strain E2 isolated from the urine specimen from a female patient in South Brazil. The genome assembly has 5,081,209 bp, a G+C content of 50.57%, and virulence factors associated with both enteroaggregative and uropathogenic *E. coli* strains.

## GENOME ANNOUNCEMENT

*Escherichia coli* is the most common uropathogen isolated from urinary tract infections (UTIs). This member of the *Enterobacteriaceae* family colonizes the gastrointestinal tract as commensal microbiota and protects against other enteropathogens (1). However, extraintestinal *E. coli* infection, mainly UTI, is a public health problem due to high prevalence, antimicrobial resistance, and health care costs. Although *E. coli* species are well studied, knowledge about this species is continually changing (2).

Uropathogenic *E. coli* (UPEC) strains are examples of opportunistic pathogens. The genome plasticity of UPEC strains increases the pathogenic potential of these bacteria, represented by virulence-specific adaptation mechanisms, such as horizontal gene transfer and gene mutations (3).

Here, we present a draft genome sequence of the *E. coli* E2 strain, isolated from a uroculture from young woman, who is 19 years old, in a tertiary hospital in South Brazil.

Genomic DNA from *E. coli* E2 was extracted with the Wizard genomic DNA purification kit (Promega). After DNA quantification by the Qubit double-stranded DNA (dsDNA) high-sensitivity (HS) assay kit, according to the manufacturer’s instructions (Life Technologies), the library was generated using the Nextera XT DNA sample preparation kit and Nextera XT index primers (Illumina). The genome sequence was determined using shotgun sequencing (MiSeq reagent kit version 2 with 300 cycles, paired-end read length of 2 × 150 bp) on an Illumina MiSeq platform. The quality metrics of reads was performed by FastQC (http://www.bioinformatics.babraham.ac.uk/projects/fastqc/), which revealed a total of 767,716 reads and quality scores >30 across all bases. Preprocessing of reads was performed with FASTX-Toolkit (http://hannonlab.cshl.edu/fastx_toolkit/index.html), removing sequencing artifacts. A shell script command was carried out to *de novo* assembly using the following assemblers: ABySS (4), SPAdes (5), SOAP*denovo*2 (6), and Velvet (7), with k-mer sequence lengths ranging from 20 to 63. The best k-mer was 47-mer for ABySS. The genome sequence was annotated via NCBI Prokaryotic Genome Annotation Pipeline (8), consisting of 222 contigs (5,081,209 bases), a G+C content of 50.57%, *N*_50_ of 74,212 bases, 4,746 protein-coding sequences (CDSs), 51 tRNAs, and five rRNAs. The genome coverage was 45×.

Two plasmids were predicted using the PlasmidFinder (9): conjugative *Escherichia coli* K-12 plasmid F, and pRSF1010_SL1344 from *Salmonella enterica* subsp. *enterica* serovar Typhimurium (accession numbers AP001918 and HE654726, respectively). Three genes that confer sulfonamide and trimethoprim resistance (*sul1*, *sul2*, and *dfrA1*, respectively) were found using ResFinder (10). In addition, the phenotype of beta-lactam resistance using ampicillin was confirmed by the presence of the *bla*_TEM-1B_ gene encoding beta-lactamase. VirulenceFinder (11) found genes related to aggregative adhesion fimbria (AAF) type III (*agg3RABCD*), which is typical of enteroaggregative *Escherichia coli* (12–14). This pathotype is suggested by the presence of diagnostic gene of dispersin transporter (*aatA*) (15). However, E2 strain carries typical UPEC virulence factors, such as secreted autotransporter toxin (*sat*) (16). These results suggest that the *E. coli* E2 strain shows the ability to colonize different host niches and has genome plasticity, as demonstrated in a previous study (17).

### Accession number(s).

This whole-genome shotgun project has been deposited at DDBJ/ENA/GenBank under the accession no. MBRL00000000. The version described in this paper is version MBRL01000000.
